# Using Real‐World Data to Guide Ustekinumab Dosing Strategies for Psoriasis: A Prospective Pharmacokinetic‐Pharmacodynamic Study

**DOI:** 10.1111/cts.12725

**Published:** 2020-01-29

**Authors:** Shan Pan, Teresa Tsakok, Nick Dand, Dagan O. Lonsdale, Floris C. Loeff, Karien Bloem, Annick de Vries, David Baudry, Michael Duckworth, Satveer Mahil, Angela Pushpa‐Rajah, Alice Russell, Ali Alsharqi, Gabrielle Becher, Ruth Murphy, Shyamal Wahie, Andrew Wright, Christopher E.M. Griffiths, Nick J. Reynolds, Jonathan Barker, Richard B. Warren, A. David Burden, Theo Rispens, Joseph F. Standing, Catherine H. Smith, on behalf of the BADBIR Study Group, the BSTOP Study Group, the PSORT Consortium, Marilyn Benham, Ian Evans, Sagair Hussain, Brian Kirby, Linda Lawson, Kayleigh Mason, Kathleen McElhone, Anthony Ormerod, Caroline Owen, Michael R. Barnes, Paola Di Meglio, Richard Emsley, Andrea Evans, Katherine Payne

**Affiliations:** ^1^ St. John's Institute of Dermatology Guy's and St Thomas' NHS Foundation Trust London UK; ^2^ St. John's Institute of Dermatology School of Basic & Medical Biosciences Faculty of Life Sciences & Medicine King's College London London UK; ^3^ Department of Medical & Molecular Genetics School of Basic & Medical Biosciences Faculty of Life Sciences & Medicine King's College London London UK; ^4^ Institute of Infection and Immunity St. George's, University of London London UK; ^5^ Department of Immunopathology Sanquin Research and Landsteiner Laboratory Amsterdam The Netherlands; ^6^ Biologics Lab Sanquin Diagnostic Services Amsterdam The Netherlands; ^7^ Dermatology Department Royal Liverpool and Broadgreen University Hospital Trust Liverpool UK; ^8^ West Glasgow Ambulatory Care Hospital Glasgow UK; ^9^ Department of Dermatology Queens Medical Centre Nottingham University Teaching Hospitals Nottingham UK; ^10^ Dermatology Department University Hospital of North Durham Durham UK; ^11^ Centre for Skin Sciences University of Bradford Bradford UK; ^12^ Dermatology Centre Salford Royal National Health Service Foundation Trust Manchester UK; ^13^ The University of Manchester, Manchester Academic Health Science Centre, National Institute for Health Research Manchester Biomedical Research Centre Manchester UK; ^14^ Dermatological Sciences Institute of Cellular Medicine Medical School Newcastle University Newcastle upon Tyne UK; ^15^ Department of Dermatology Royal Victoria Infirmary Newcastle Hospitals NHS Foundation Trust Newcastle upon Tyne UK; ^16^ Institute of Infection, Immunity and Inflammation University of Glasgow Glasgow UK; ^17^ Infection, Immunity, Inflammation Section UCL Great Ormond Street Institute of Child Health London UK

## Abstract

Variation in response to biologic therapy for inflammatory diseases, such as psoriasis, is partly driven by variation in drug exposure. Real‐world psoriasis data were used to develop a pharmacokinetic/pharmacodynamic (PK/PD) model for the first‐line therapeutic antibody ustekinumab. The impact of differing dosing strategies on response was explored. Data were collected from a UK prospective multicenter observational cohort (491 patients on ustekinumab monotherapy, drug levels, and anti‐drug antibody measurements on 797 serum samples, 1,590 measurements of Psoriasis Area Severity Index (PASI)). Ustekinumab PKs were described with a linear one‐compartment model. A maximum effect (E_max_) model inhibited progression of psoriatic skin lesions in the turnover PD mechanism describing PASI evolution while on treatment. A mixture model on half‐maximal effective concentration identified a potential nonresponder group, with simulations suggesting that, in future, the model could be incorporated into a Bayesian therapeutic drug monitoring “dashboard” to individualize dosing and improve treatment outcomes.

**Study Highlights**

**WHAT IS THE CURRENT KNOWLEDGE ON THE TOPIC?**

☑ There is significant variation in response to biologic therapy in immune‐mediated diseases, some of which is driven by differences in drug exposure. Ustekinumab is a monoclonal antibody targeting the p40 subunit common to IL‐12/23, and is widely used in the treatment of psoriasis and inflammatory bowel disease. Studies investigating therapeutic drug monitoring and dose individualization for ustekinumab are limited.

**WHAT QUESTION DID THIS STUDY ADDRESS?**

☑ Can pharmacokinetic/pharmacodynamic (PK/PD) modeling define dose adjustments that will improve outcomes in patients with psoriasis treated with ustekinumab?

**WHAT DOES THIS STUDY ADD TO OUR KNOWLEDGE?**

☑ Our PK/PD model reinforces findings from phase III clinical trials, and we additionally characterize a mixed distribution of half‐maximal effective concentration that could identify responder and nonresponder subgroups. Model simulations suggest that dose escalation/interval reduction may benefit partial responders.

**HOW MIGHT THIS CHANGE CLINICAL PHARMACOLOGY OR TRANSLATIONAL SCIENCE?**

☑ Incorporating these findings into a Bayesian therapeutic monitoring algorithm could facilitate individualized ustekinumab dosing, including identifying nonresponders for early switching. These findings may be generalizable to other disease settings.

The advent of biologic therapies means that complete disease remission is now achievable in patients with immune‐mediated inflammatory diseases, including psoriasis, rheumatoid arthritis, and inflammatory bowel disease. Nevertheless, poor response or loss of response remains a significant problem for many,^1^, ^2^, ^3^ and is at least partly explained by differences in drug exposure. This, in turn, is influenced by treatment adherence and pharmacokinetic (PK) factors, including bodyweight and the development of anti‐drug antibodies (ADA). Interest has, therefore, centered on therapeutic drug monitoring (TDM) to guide dosing for individual patients in an adaptive and timely fashion, and potentially to reduce clinical costs.^4^, ^5^, ^6^

Broadly speaking, TDM strategies advocate population‐based target trough concentrations for dose adjustment, using a reactive rather than proactive approach.^7^, ^8^, ^9^ Integration of pharmacodynamic (PD) outcomes to yield PK/PD models is rare, but these could feasibly be included in Bayesian prediction algorithms to predict and adjust dosing strategy on an individual level.^10^, ^11^ To date, investigation of the effectiveness and utility of TDM has largely been confined to tumor necrosis factor inhibitors, the first of many cytokine‐targeted biologic therapies in immune‐mediated inflammatory diseases.

Ustekinumab, a highly effective biologic targeting the IL23‐Th17 canonical pathway, is a fully human immunoglobulin G1 kappa monoclonal antibody binding to the p40 subunit shared by IL‐12 and IL‐23. Initially developed for psoriasis^12^ (where it remains first‐line), it is now also licensed for use in psoriatic arthritis and inflammatory bowel disease.^13^, ^14^ Studies investigating the relationship between ustekinumab exposure and outcome are few, generally limited to descriptive or empirical analyses, and report mixed results.^15^, ^16^, ^17^, ^18^, ^19^ Understanding exposure‐response is complicated by the fact that some patients’ disease may not respond to IL‐23‐Th17 therapies, and furthermore, some may receive subtherapeutic drug exposure due to PK variability.

Ustekinumab dosing for psoriasis comprises a fixed dose (45 mg/90 mg) stratified by bodyweight (less/more than 100 kg, respectively) given subcutaneously at week 0, week 4, and then 12‐weekly. Real‐world data show that those with higher baseline body mass index are less likely to respond,^20^ and more likely to need higher cumulative doses over the first year of treatment,^21^ suggesting that a proportion of patients may have insufficient drug exposure. On the other hand, a recent phase IIIb study reports a subset of patients in who complete response was maintained, despite lengthening the dosing interval.^22^

Psoriasis represents an ideal disease model to investigate the utility of TDM,^6^, ^23^ because treatment response is visually observed and easily quantifiable over time. Here, we use a large‐scale real‐world data set from the multicenter cohort study Biomarkers of Systemic Treatment Outcomes in Psoriasis (BSTOP), within the UK pharmacovigilance registry British Association of Dermatologists Biologics and Immunomodulators Registry (BADBIR). This resource captures deep clinical phenotyping, including serum ustekinumab sampling and ADA measurements, along with repeated, longitudinal measures of clinical severity using the validated tool known as the Psoriasis Area and Severity Index (PASI).^24^ The PASI score ranges from 0‐72, and the usual biologic treatment eligibility threshold is ≥ 10. Response is judged as the percentage reduction in PASI from baseline (e.g., “PASI75” means a 75% decrease in PASI).

As a first step to exploring the clinical potential of a Bayesian therapeutic monitoring algorithm for ustekinumab, we applied PK/PD modelling to this real‐world data set to investigate the relationship between ustekinumab exposure and treatment response, and compared our findings to published PK and PD models derived from phase III clinical trials.^25^, ^26^ We also simulated the impact of alternative dosing regimens on response, and explored the clinical utility of early assessment of trough concentration and PASI change from baseline.

## Materials And Methods

### Patient and data

#### Ethics approval

This study was conducted in the spirit of the 1996 International Conference on Harmonisation in Good Clinical Practice (ICH‐GCP) 1996, and in accordance with the 2008 Declaration of Helsinki. Two studies provided samples and data within the Psoriasis Stratification to Optimise Relevant Therapy (PSORT) consortium, aiming to understand determinants of response to biologics: BSTOP (approved by The South East London REC 2 Ethics Committee, 11/H0802/7), and its nested study PSORTD (PSORT Discovery; approved by the National Research Ethics Service Committee London – London Bridge, 14/LO/1685). Written informed consent was obtained from all subjects prior to enrollment.

### Patients and setting

BSTOP is a prospective multicenter (*n* = 60) observational study, established in 2011 following a 2009 pilot, aiming to identify markers of outcomes to systemic therapies in psoriasis. All UK adults fulfilling BSTOP inclusion criteria^27^ and enrolled onto BADBIR^28^ were invited to participate. BADBIR has recruited > 18,000 patients since 2007, and includes patients with: dermatologist‐diagnosed psoriasis; age > 16 years; and started on, or switched to a conventional systemic therapy or a biologic therapy within the previous 6 months. Detailed information is recorded, including demographics, comorbidities, treatments, and adverse effects. Clinical response is assessed longitudinally using the gold standard assessment tool PASI.^24^ PSORTD is a nested study with the same inclusion criteria, embedded within the BSTOP clinical site network (11/79 centers), collecting samples at rigorously defined time points within the first 3 months of treatment. For the current study, inclusion criteria were: patients enrolled onto BSTOP and/or PSORTD, taking ustekinumab monotherapy, and with ≥ 1 serum sample and ≥ 1 recorded PASI within a year after starting treatment.

### Drug level and ADA measurements

Venous blood samples were collected between June 2009 and December 2016 during routine clinic visits. All samples were centrifuged at 2,000 g for 10 minutes and serum aliquots frozen (−80°C). In this pragmatic study, samples were not collected from every patient at every time point, and most were taken without reference to treatment administration (i.e., trough/non‐trough not specified). The ustekinumab assay was an enzyme‐linked immunosorbent assay^29^ using IL‐12 as a target to capture ustekinumab with a lower limit of detection 0.02 µg/mL. ADA were measured using a radioimmunoassay^30^ with a positive cutoff at 12 arbitrary units/mL. If several ADA measurements were available for the same patient, only the measurement with the highest titer was included for analysis.

### Clinical outcome measures

Psoriasis severity was measured using PASI.^24^ PASI score at 6 months was used to categorize patients into three responder types: full responders achieving ≥ 75% PASI decrease from baseline (PASI75); partial‐responders ≥ 50% and < 75% decrease from baseline (PASI50‐75); and nonresponders < 50% decrease from baseline (PASI < 50).

### Ustekinumab real‐world PKs/PDs

A sequential population PK then PD model was developed using nonlinear mixed‐effects modeling in NONMEM (version 7.3).^31^ The PK model included all patients, whereas the PD model was developed on the subset of patients with baseline PASI ≥ 10, because current treatment guidelines restrict biologic therapy to patients with a PASI ≥ 10.

The PK structural model was one‐compartment with first‐order subcutaneous absorption and first‐order elimination (see **Supplementary Materials** for the final structural model and NONMEM code). Allometric scaling weight scaling centered on 70 kg was added *a priori* with an exponent of 0.75 on clearance and 1 on volume.^32^

A turnover model was used to describe PASI evolution over time (see **Supplementary Materials** for the final structural model and NONMEM code). Drug effect was via a maximum effect (E_max_) model inhibiting the *K*_in_ parameter (i.e., development/progression of psoriasis skin lesions). Upon finding a possible bimodal distribution on half‐maximal effective concentration (EC_50_), a mixture model using the NONMEM $MIXTURE regime was used.^33^

### Covariate selection and model evaluation

Baseline demographics (age, sex, and ethnicity), alcohol and smoking status, anthropometric measures (body mass index and waist circumference), psoriasis characteristics (disease duration, involvement of palms/soles, and psoriatic arthritis), and comorbidities were tested using the Stepwise Covariate Model‐building method^34^ with forward selection (*P* = 0.05) and backward elimination (*P* = 0.01) based on the likelihood ratio test.^35^ Only covariates recorded in ≥ 10% of patients were tested. For missing covariates, the missing values of continuous covariates were replaced with the medians, and the missing values of categorical covariates were replaced with the most common numerical categories of individual covariates.^34^

Model fit was assessed using plots of observations vs. model predictions, standardized residuals, likelihood‐based diagnostics (via the NONMEM objective function value), and assessment of model simulation properties via the visual predictive check (VPC).^36^ Plots were created using R (R Core Team. R: A language and environment for statistical computing. Vienna, Austria: R Foundation for Statistical Computing; 2019).

### Simulation of dose adjustment

Guidelines suggest that ustekinumab is stopped if PASI75 has not been achieved by 16 weeks.^12^ We therefore explored whether switching to alternative dosing regimens, after the initial 16 weeks of standard treatment, may impact the probability of achieving PASI75/PASI90 (clear/almost clear on the Physician's Global Assessment). Specifically, we simulated 1,000 patients (63% full‐responders, 23% partial‐responders, and 14% nonresponders; i.e., the same proportions as observed in the cohort) for two dosing groups 45 mg and 90 mg, taking into account individual variability and significant PK covariates. Beyond 16 weeks, we simulated two potential alternative dosing frequencies (i.e., 8‐weekly and 16‐weekly, alongside the standard 12‐weekly regimen). To assess change in dosing level, we also performed simulations using covariates from the patient group receiving 45 mg, but with an administered dose of 90 mg.

### Identifying a target trough concentration for response at 6 months

To determine an early target trough concentration predictive of an 80% probability of longer‐term response (as defined by PASI75 at 6 months), 1,000 patients were simulated based on parameter estimates in the mixture 1 subpopulation. This was performed separately for both 45 mg and 90 mg doses on the standard regimen. Individual variability and significant PK covariates were taken into account during simulation. The trough concentrations at week 4 (median and 95% prediction interval) were then derived for an 80% probability of PASI75 at 6 months.

Individual predictions of ustekinumab trough concentration at week 4 were plotted against the observed PASI change from baseline by 4 weeks for all patients to determine whether early response and trough concentration were related to 6‐month outcome.

## Results

### Patients and data

Within a prospective multicenter observational study (60 dermatology centers across the United Kingdom), we identified 491 patients with psoriasis on ustekinumab monotherapy fulfilling our inclusion criteria*.* Ustekinumab drug levels and ADA measurements were derived from 797 serum samples, and 1,590 PASI measurements were available over the first year of treatment. The percentages of subjects with 1/2/3/4/5 serum samples were 58.9/22.8/15.9/2.0/0.4, respectively. Of 491 patients, there were 239 responders, 107 partial‐responders, 131 nonresponders, and 14 with unknown response. Of note, the average number of serum samples per patient was similar between responders and nonresponders (1.7 vs. 1.6, respectively).

The PK model included all patients’ PASI measurements, whereas the PD model included 348 patients and 1,136 PASI measurements. PD results from all 491 patients can be found in **Table** **S1** (**Supplementary Materials**).

Baseline characteristics of the cohort (**Table** 1) were consistent with the psoriasis population prescribed biologic therapy: mostly men (65.2%), with severe longstanding disease (median PASI 12.1; median disease duration 21.0 years), and a comorbidity profile dominated by features of the metabolic syndrome (median weight 91.8 kg, diabetes mellitus 12.6%, and hypertension 28.7%). There were 40.9% of patients who were biologic‐naïve, and 57.4% were on 45 mg ustekinumab vs. 42.6% on 90 mg. No patients were recorded to have changed dose or dosing interval during the first year of treatment.

**Table 1:** Summary of baseline variables for all patients (*n* = 491), including demographics, disease characteristics, and comorbidity burden

Variable	Median or percentage	Range	Missing (%)
No. of serum measures per subject	1.6	(1–5)	–
No. of PASI measures per subject	3.2	(1–8)	–
No. of ustekinumab doses per subject	6	(1–6)	–
Duration of therapy per subject (year)	1	(0–1)	–
Sex (male, %)	65.2	–	0.0
Age (year)	45.5	(18.9–81.2)	0.0
Ethnicity (white, %)	85.7	–	0.0
Weight (kg)	91.8	(48.2–174)	11.4
BMI (kg/m^2^)	30.5	(18.0–60.2)	13.0
Waist (cm)	104	(65–161)	14.5
Alcohol (yes, %)	63.3	–	3.9
Smoking (yes, %)	25.1	–	3.9
Baseline PASI	12.1	(0–39.7)	24.2
Disease duration (year)	21.0	(1.0–68)	8.8
Palms/sole involvement (yes, %)	18.9	–	10.2
Inflammatory arthritis (yes, %)	22.2	–	7.3
Biologic‐naïve^a^ (yes, %)	40.9	–	0.0
Comorbidity (yes, %) (AS/MDD/DM/DLP/HT/LD)	11.2/17.3/12.6/10.0/28.7/10.0	–	3.7/3.7/3.7/3.7/3.7/3.7
Creatinine (μmol/L)	78	(46–403)	36.3

Median or percentage: median values for continuous variables or percentage for categorical variables, missing (%): the percentage of records for a variable not available.

AS, asthma; BMI, body mass index; DLP, dyslipidemia; DM, diabetes mellitus; HT, hypertension; LD, liver disease; MDD, major depressive disorder; PASI, Psoriasis Area and Severity Index.

^a^ Five of 491 (1.0%) patients had received ustekinumab prior to the study period.

### Ustekinumab real‐world PK

As expected, ustekinumab PKs were linear,^37^ and adequately described using a one‐compartment model. Parameter estimates are summarized in **Table** 2. The absorption rate constant, apparent clearance, and volume of distribution (0.23 day^−1^, 0.44 L/day, and 10.2 L) were similar to those reported in ustekinumab phase III development (0.35 day^−1^, 0.47 L/day, and 15.7 L).^25^

**Table 2:** Parameter estimates from the ustekinumab PK model for all patients (*n* = 491)

Parameter (unit)	Estimate	RSE (%)
k_a_ (/day)	0.23	16.1
CL/F (L/day)	0.44	6.7
V/F (L)	10.2	8.2
BSV on CL (%)	44.7	10.3
BSV on V (%)	36.5	28.9
corr _CL and V_	0.37	21.1
coeff_weight on CL_	0.75 (fix)	–
coeff_weight on V_	1 (fix)	–
coeff_bionaïve on CL_	−0.13	34.1
coeff_creatinine on CL_	−0.36	35.5
coeff_waist on CL_	0.84	18.0
coeff_alcohol on CL_	0.15	42.7
Proportional error (%)	61.3	3.2
Additive error (SD)	0.007	13.7

BSV, between‐subject variability; CL/F, apparent clearance; coeff, coefficient of a covariate on CL or V; corr, correlation coefficient between CL and V; k_a_, absorption rate constant of ustekinumab; PK, pharmacokinetic; RSE, relative standard error; SD, standard deviation; V/F, apparent volume.

**Figure 1:**
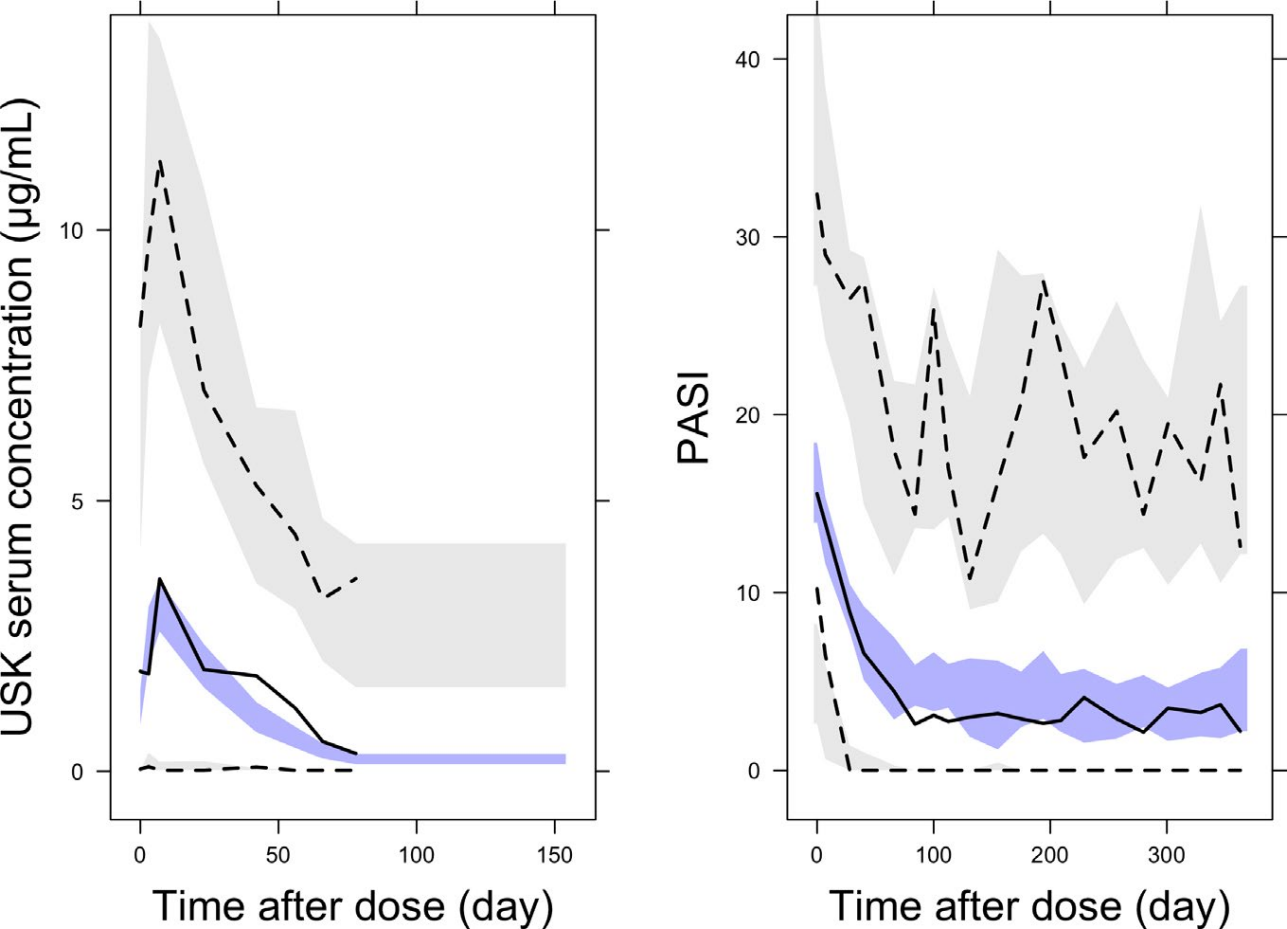
Visual predictive check for ustekinumab pharmacokinetic model (left), and pharmacodynamic mixture model (right). The 95% prediction intervals are shown for predicted data in grey (97.5th and 2.5th percentiles top and bottom, respectively) and blue (50th percentile) bands with corresponding observed percentiles shown as broken black (97.5th and 2.5th percentiles top and bottom, respectively) and solid black (50th percentile) lines. PASI, Psoriasis Area Severity Index; USK, ustekinumab.

Between‐subject variability (BSV) was 44.7% and 36.5% for clearance and volume of distribution, respectively. Relative SE values ranged from 6.7% to 42.7%. A VPC (**Figure** 1, left panel) indicated that the PK model adequately described the serum ustekinumab concentration over time.

In addition to *a priori* allometric weight scaling, waist circumference and alcohol consumption were associated with increased apparent clearance (*P* < 0.01), whereas increased creatinine and biologic‐naïve status had the opposite effect (*P* < 0.01). Of note, when waist circumference was excluded from the model, diabetes status became a significant covariate (data not shown; diabetes status not included in the final model). In patients with measurable ADA (*n* = 16), clearance was higher than in those with nonmeasurable ADA (*P *> 0.05) and increased with increasing ADA level (**Figure** **S2** in **Supplementary Materials**).

### Mixture modeling separates full‐responders and nonresponders in the PD model

**Figure 2:**
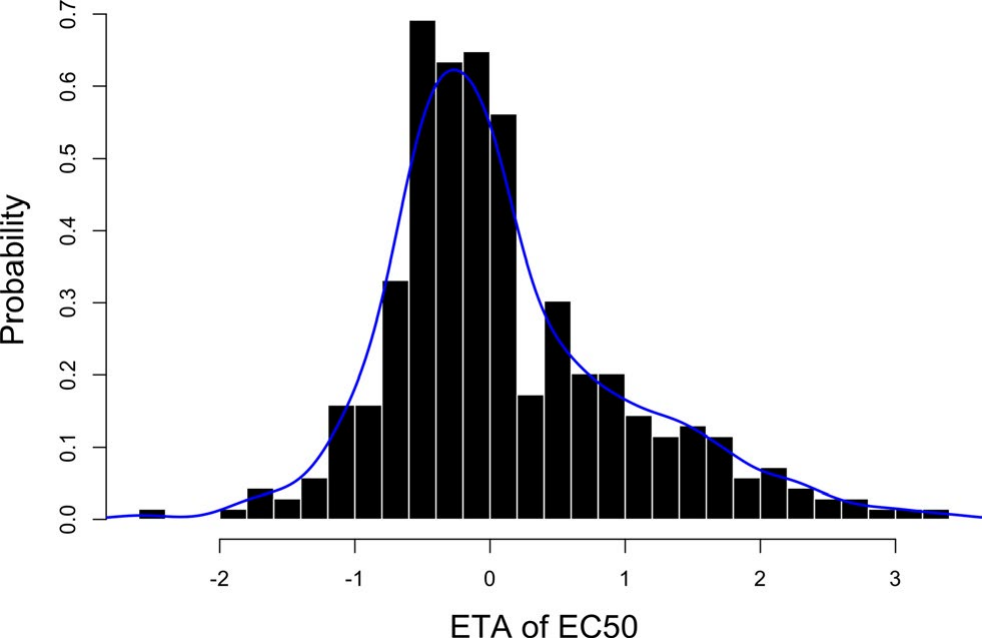
Distribution of random effects (ETA) of EC50 in the single population model. Solid blue line: density of the distribution. EC50, concentration of ustekinumab giving 50% of the maximum IL‐12/IL‐23 inhibition.

**Table** 3 summarizes the PD model parameter estimates. The estimated EC_50_ (concentration of ustekinumab giving 50% of the maximum IL‐12/IL‐23 inhibition) was 0.14 μg/mL, with large BSV (148.3%), which was not associated with any of the covariates using either manual or automatic selection methods. The BSV had an asymmetric bimodal distribution (**Figure** 2), and inclusion of a bimodal distribution on EC_50_ using $MIXTURE significantly improved model fit. The two subpopulations comprised 76.2% and 23.8% of patients, respectively. Mixture group 1 had a typical EC_50_ of 0.07 μg/mL, whereas in mixture group 2 the EC_50_ was 1.21 μg/mL (**Table** 3). A VPC plot (**Figure** 1, right panel) indicated that the PK/PD model adequately described the PASI change from baseline over time.

**Table 3:** Parameter estimates for patients with baseline PASI ≥ 10 (*n* = 348) using two different ustekinumab PD models

Parameter (unit)	Estimate from single model (RSE%)	Estimate from mixture model (RSE%)
Baseline PASI	15.5 (4.4)	15.8 (4.2)
*k*_out_ (/day)	0.02 (6.9)	0.02 (7.3)
E_max_	1 (fix)	1 (fix)
EC_50_ (μg/mL)	0.14 (15.0)	Mixture 1: 0.07 (17.3)
		Mixture 2: 1.21 (22.2)
BSV on baseline (%)	43.6 (7.3)	41.4 (7.6)
BSV on *k*_out_ (%)	66.4 (7.9)	66.9 (8.8)
BSV on EC_50_ (%)	148.3 (9.5)	42.7 (58.2)
Additive error (SD)	3.3 (1.7)	3.3 (1.7)
delta OBJV	–	−12.2

Single model: patients treated as from a single population, mixture model: automatic stratification into two subpopulations using $MIXTURE.

BSV, between‐subject variability; , delta OBJV, objective function value change from single model; EC_50_, concentration at 50% of maximum inhibition on IL 12 and 23; E_max_, maximum inhibition effect of ustekinumab; *k*_out_, elimination rate constant of skin lesions; PASI, Psoriasis Area Severity Index; PD, pharmacodynamics; RSE, relative standard error; SD, standard deviation.

Full‐responders: ≥ 75% reduction from baseline; partial‐responders: ≥ 50% and < 75% reduction from baseline; nonresponders: < 50% reduction from baseline.

Comparing the mixture model classification with the actual observed response rate at 6 months, mixture group 1 comprised 68.6% who achieved PASI75 (full‐responders), 24.0% who achieved PASI50‐75 (partial‐responders), and 7.4% who achieved PASI < 50 (nonresponders). By contrast, mixture group 2 comprised only 5.4% full‐responders, 23.2% partial‐responders, and 71.4% nonresponders. Most full‐responders, therefore, appeared in mixture group 1 (low EC_50_), whereas most nonresponders appeared in mixture group 2 (high EC_50_). There was an even proportion of partial responders in each group, but further $MIXTURE stratification into three subpopulations did not improve model fit.

### Simulations to explore alternative ustekinumab dose regimens

**Figure 3:**
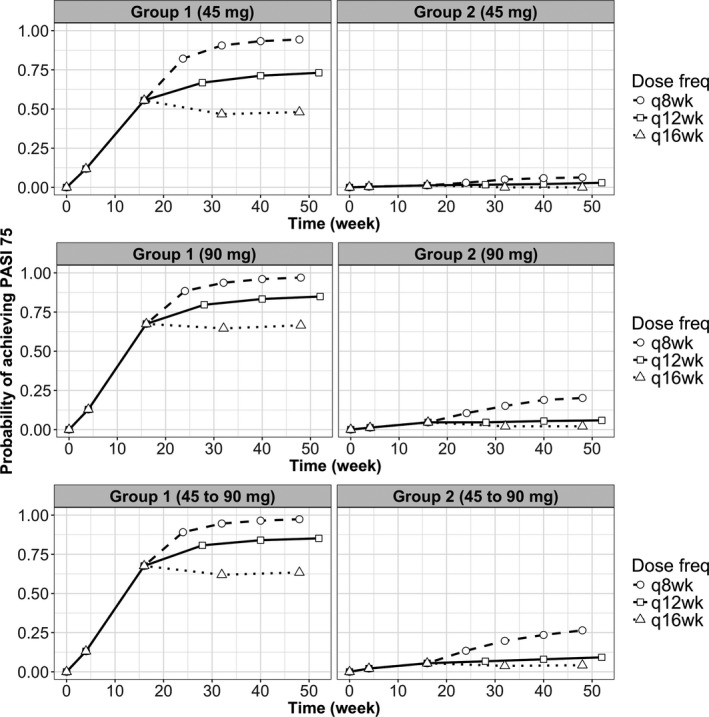
Simulated profiles using the mixture model of the probability of achieving PASI75 after 8 weekly, 12 weekly, and 16 weekly ustekinumab injections in the patient group on 45 mg (top), on 90 mg (middle), and simulating a 90 mg dose using parameters from the patient group on 45 mg (bottom) (Group 1: using parameters from mixture group 1, Group 2: using parameters from the mixture group 2). PASI, Psoriasis Area Severity Index.

We used the final parameter estimates from the mixture model (with EC_50_ values consistent with being in mixture groups 1 and 2, respectively), to explore the impact of alternative dosing regimens on response outcomes (**Figure** 3). Simulated profiles for the standard dose and interval (12‐weekly) showed a sustained response over time, and a higher probability of achieving PASI75 for patients on 90 mg compared with 45 mg overall. For patients in mixture group 1, the probability of achieving PASI75 after 1 year of treatment could be increased toward 100% by reducing the dosing interval from 12‐weekly to 8‐weekly. For patients in mixture group 2, the probability of achieving PASI75 could only be substantially increased in patients on 45 mg, and only to around 20%. None of these simulated interventions seemed to impact on the probability of achieving PASI90 (**Figure** **S3**, right‐hand panel).

To explore whether increasing the dosing interval may be possible in patients with good response, simulation using a typical EC_50_ from mixture group 1 subpopulation (mainly full‐responders) suggested that the probability of achieving PASI75 dropped from near 75% to below 50% when the dosing interval changed from 12‐weekly to 16‐weekly, with a substantial decrease also seen in the 90 mg group (**Figure** 3, left‐hand panel). A similar pattern was seen for the probability of achieving PASI90 (**Figure** **S3** in **Supplementary Materials**, left‐hand panel).

To explore whether increasing the ustekinumab dose may improve response, we also simulated a 90 mg dose using parameters from the patient group on 45 mg (**Figure** 3, bottom panel). This suggested that in patients on 45 mg, increasing the dose to 90 mg is less likely to improve response than reducing the dosing interval from 12‐weekly to 8‐weekly.

### Toward early stratification of subsequent response: Early trough concentration and initial PASI reduction from baseline

**Figure 4:**
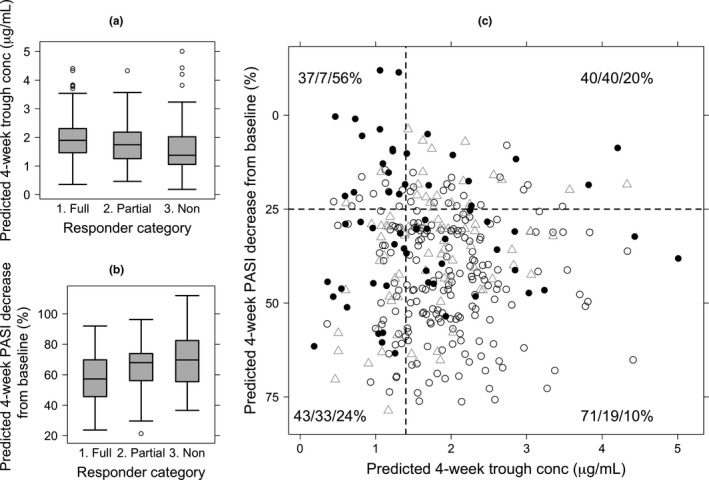
Individual predicted 4‐week PASI change from baseline vs. 4‐week ustekinumab trough concentration. (**a**) Predicted 4‐week trough concentration by responder category; (**b**) predicted 4‐week PASI decrease from baseline (%) by responder category; (**c**) predicted 4‐week PASI decrease from baseline (%) vs. predicted 4‐week trough concentration. In **c**, open circles represent patients who were full‐responders at 6 months, triangles are partial‐responders, and filled circles are nonresponders. Horizontal and vertical lines represent 4‐week PASI cutoff of 25% decrease and 4‐week trough concentration of 1.4 μg/mL, respectively. The numbers in each quadrant represent the percentage of 6‐month full‐responder, partial‐responder, and nonresponders in each quadrant, respectively. PASI, Psoriasis Area Severity Index.

The licensed first dose interval of ustekinumab is 4 weeks to create a loading dose effect; therefore, week 4, immediately prior to the second dose, represents a possible early intervention point. A target of 1.4 mg/L (expected variability 0.4–3.4 μg/mL) was identified as the median 4‐week drug concentration if the dose was adjusted to project an 80% response rate at 6 months for the mixture 1 subpopulation (**Figure** 4). Based on this qualitative interpretation of **Figure** 4, we next performed exploratory analysis using individual predictions of ustekinumab concentrations and PASI response.

In the subset of observed patients with PASI ≥ 10, individualized predictions of 4‐week ustekinumab trough concentration and 4‐week change from baseline PASI were evaluated with respect to three 6‐month responder categories: full (PASI75), partial (PASI50‐75), and nonresponder (PASI < 50). Responders tended to have higher trough concentrations and large initial PASI reduction from baseline, whereas nonresponders demonstrated low trough concentration and small initial PASI reduction (**Figure** 4 **a,b**). We therefore divided simulated patients into four groups based on a trough concentration cutoff of 1.4 μg/mL (as above), with a pragmatically selected 4‐week PASI25 (**Figure** 4 **c**). The subsequent 6‐month response profiles for each group imply that 4‐week trough concentration could be combined with initial PASI trajectory to begin to stratify probable responder phenotype.

## Discussion

### Key findings

Using psoriasis as a disease model, we present the first real‐world study investigating the PK/PD relationship between serum ustekinumab levels and treatment response. PK/PD findings from our study support those reported from clinical trials, although we were able to identify two responder subpopulations in our PD mixture model, whereas this was not reported in the trial data. Our mixture model suggests that there are two subpopulations of low and high EC_50_ values, which largely separate patients achieving 6‐month PASI75 (full‐responders) and patients failing to achieve PASI50 (nonresponders). Within the high EC_50_ group, there are likely to be a subgroup of true nonresponders for whom no amount of ustekinumab will be effective. However, our exploratory simulations suggest that dose escalation may improve probability of response toward 100% in patients already achieving at least PASI50‐75 (partial‐responders), whereas this approach does not seem to substantially improve probability of response in nonresponders (**Figure** 3). At 4 weeks, combined trough ustekinumab concentration and change in PASI could be used as a guide to determine likely clinical outcome at 6 months (**Figure** 4).

### The PK/PD model

Our ustekinumab PK findings are largely consistent with those estimated from clinical trial data, including absorption rate constant, clearance, and volume of distribution.^25^ A broadly similar approach to covariate selection was adopted to that taken by Zhu *et al*.^25^, but also included factors known to influence response to biologics across the BADBIR cohort.^38^ Covariates that we found to be significantly associated with clearance overlapped with those from the published model.^25^ Of note, Zhu *et al*.^25^ reported a positive relationship between creatinine clearance and drug clearance, and in line with this we found increased serum creatinine concentration to be associated with reduced drug clearance (creatinine clearance values were not derived in our study). Because ustekinumab is not primarily cleared via the kidneys, this trend is unexpected and possibly indicates that creatinine may act as a proxy for some other influence on ustekinumab clearance, such as an unmeasured comorbidity. Zhu *et al*.^25^ additionally reported that diabetes, albumin level, and alkaline phosphatase level significantly affected clearance. Although the latter two measurements were unavailable in our cohort, we also found clearance to be around 30% higher in diabetic vs. nondiabetic patients. Possible factors accounting for this include decreased lymphatic function, increased glycation of ustekinumab, and differences in body composition.^38^, ^39^ However, diabetes was not included in the final model as its effect was accounted for by waist circumference. Although we found higher bodyweight with *a priori* allometry to be the only covariate associated with increased volume of distribution, Zhu *et al*.^25^ additionally reported that diabetes and ethnicity were significant covariates. Clearance was increased in the small number of patients in whom ADA were detected, reflecting the likely mechanism that complexes formed between ADA and therapeutic antibodies trigger immune processes of internalization and lysosomal degradation.^40^

Regarding the PD model and considering the chronic nature of psoriasis, we assumed a turnover model reflecting the dynamics of progression and remission of skin lesions over time. The turnover half‐life of skin lesions was estimated to be ~ 5 weeks, similar to the reported value used for the PD model derived from clinical trial data^26^ (34.7 days vs. 22.1 days). Psoriasis turnover half‐life (*k*_out_) was consistent for both (single and mixture) PD models (**Table** 3), and also for models that included all patients regardless of baseline PASI (**Table** **S1**). This implies that the turnover model captures the underlying disease trajectory, and is robust to changes in the drug effect model and baseline disease severity. In our study, EC_50_ was found to have a bimodal distribution, and unlike Zhou *et al*.’s study,^26^ a significant improvement in model fit was obtained with the mixture model. Based on the individual probabilities in the mixture model, EC_50_ estimates for the two subpopulations differed by ~ 20‐fold. Because most responsive patients were in the low EC_50_ group and most nonresponsive patients were in the high EC_50_ group, this may imply that a Bayesian model taking into account patient covariates, initial PASI trajectory, and ustekinumab concentration may help identify primary nonresponders and guide dose adjustments in partial responders. Further automatic stratification into three subpopulations was unsuccessful, with no improvement in model fit or identification of three distinctive subgroups. This might be due to an insufficiently strong signal of PASI change over time, in particular within the nonresponder subgroup.

### Strengths and limitations

This is the first PK/PD study of ustekinumab that draws on real‐world clinical data, as opposed to clinical trial data. A key strength of our unique cohort is high external validity; > 50% of all UK patients with psoriasis taking biologics are registered on BADBIR, and 95% of UK dermatology centers prescribing biologics for psoriasis contribute data to BADBIR. It is, therefore, of substantial interest that key PK parameters are consistent between models derived from real‐world vs. clinical trial data. While taking the same modelling approach as Zhu *et al*.^25^ and Zhou *et al*.,^26^ we maximized clinical relevance by including covariates already known to affect response to biologics across the BADBIR cohort. As reported elsewhere, we also confirm in our real‐world cohort that ustekinumab has relatively low immunogenicity in the setting of clinical practice, with only 3.3% of patients developing detectable ADA.^23^

Our study had some limitations. As with any real‐world cohort, a key issue is missing data. For missing injection dates, we made the assumption of perfect patient compliance without changes on doses and dosing regimen during treatment course. In the United Kingdom, ustekinumab injections are almost always administered by nurses, and, therefore, nonadherence rates are low.^41^ For missing covariates, we replaced the missing values with medians or the most common categories. Unlike a clinical trial setting, there was no group of patients on placebo only, to give insight into the untreated disease trajectory. There were also unbalanced and missing PK/PD observations from certain patients or at certain time points. However, these shortcomings did not seem to impact our population modelling results and interpretation, because parameter estimates were close to those reported from phase III clinical trials.^25^, ^26^ Of note, 60% of BADBIR centers recruit to the BSTOP study, whereby patients also consent to giving biological samples, so there remains inherent potential for bias in patient selection. However, we have maximized inclusivity by not applying selection criteria for the current study cohort beyond needing ≥ 1 serum sample/PASI. Finally, our proposed clinical algorithm (**Figure** 4) is unsophisticated, and only applies to the 4‐week time point.

### Clinical implications

Using real‐world clinical data, we present a PK/PD model for patients with psoriasis on ustekinumab, demonstrate potential utility in modifying ustekinumab dosing in certain patient subsets, and show that assessing trough concentration and PASI trajectory at week 4 is informative with respect to likely 6‐month response. These findings require validation, but suggest that consideration should be given both to the initial PASI response and ustekinumab trough concentration. To expand on this specifically, although finding a trough lower than 1.4 μg/mL should generally prompt consideration of a dose increase, by simulating increased dosing frequencies we have shown that the effect of this dose increase will likely also depend on initial PASI response. For such patients a good response (> 25% decrease) at week 4 indicates a higher likelihood that partial response would have been achieved without a dose increase than does a poor 4‐week response; our simulations show that a dose increase may “convert” such cases to full response. A poor early response with high 4‐week trough levels would indicate a group with a low probability of response, and may indicate a primary nonresponder group where therapy should be switched; certainly it would seem prudent to reassess PASI response in short order (e.g., another 4 weeks). Currently, serum ustekinumab concentrations are only sporadically measured in routine clinical practice, and clinical response is not evaluated as early as 4 weeks. Our recommendations point to a need for a change in practice, and should be evaluated in a prospective dose‐intervention study.

In the future, similar models could be implemented into a Bayesian dashboard system, so that real‐time predictions of response could be used to inform dosing and treatment switching decisions.^42^ Finally, integration of PK/PD models with genetic and other omic data may help identify disease endotypes, allowing further personalization of therapy for individual patients with psoriasis.

## Funding

This work was funded by a Medical Research Council (MRC) Stratified Medicine award (MR/L011808/1). The Psoriasis Association (RG2/10), the NIHR Biomedical Research Centre at King's College London/Guy's and St. Thomas' NHS Foundation Trust, the NIHR Manchester Biomedical Research Centre, and the NIHR Newcastle Biomedical Research Centre. T.T. is supported by an MRC Clinical Research Training Fellowship (MR/R001839/1). N.D. is supported by Health Data Research UK (MR/S003126/1). N.J.R. is supported by the Newcastle MRC/EPSRC Molecular Pathology Node and the Newcastle NIHR Medtech and In vitro diagnostics Co‐operative. C.E.M.G. and N.J.R. are NIHR Senior Investigators. J.F.S. is supported by an MRC Fellowship (MR/M008665/1).

## Conflict of Interest

C.E.M.G. has received honoraria and/or research grant support (University of Manchester) from AbbVie, Almirall, Bristol Meyers Squibb, Celgene, GSK, Janssen, LEO Foundation, Lilly, Novartis, Pfizer, Sandoz, Sun Pharma, and UCB Pharma. N.J.R. has received honoraria, travel support, and/or research grants (Newcastle University) from AbbVie, Almirall, Celgene, Genentech, Janssen, Novartis, Pfizer, Sanofi Genzyme Regeneron, and UCB Pharma Ltd. J.B. has received honoraria, travel support, and/or research grants (King's College) from AbbVie, Pfizer, Novartis, Janssen, Roche, Regeneron, Lilly, UCB, Sun Pharma, Boehringer Ingelheim, and GSK. R.B.W. has received honoraria and/or research grants from AbbVie, Almirall, Amgen, Boehringer Ingelheim, Celgene, Janssen, Leo, Lilly, Novartis, Pfizer, Sanofi, Xenoport, and UCB. A.D.B. has received honoraria from AbbVie, Amgen, Boehringer Ingelheim, Celgene, Janssen, Leo, Lilly, Novartis, and Pfizer. T.R. has received honoraria for lectures from Pfizer, AbbVie, and Regeneron, and a research grant from Genmab. D.S. has received departmental research funding from AstraZeneca. C.S. has received departmental research funding from AbbVie, GSK, Pfizer, Novartis, Regeneron, and Roche. N.W. acts as statistician on a trial funded by AstraZeneca. The PSORT consortium has a number of industry partners; see http://www.psort.org.uk. All other authors declared no competing interests for this work.

## Author Contributions

S.P., T.T., N.D., J.F.S., and C.H.S. wrote the manuscript. C.H.S., J.F.S., and S.P. designed the research. All authors performed the research. S.P., T.T., N.D., J.F.S., and C.H.S. analyzed the data.

## Supporting information


**Figures S1–S7.**
**Table S1.**
**Code S1, Code S2, Code S3.**
