# Supplementary material for: Using Real‐World Data to Guide Ustekinumab Dosing Strategies for Psoriasis: A Prospective Pharmacokinetic‐Pharmacodynamic Study
Source: Clin Transl Sci. 2020 Jan 29;13(2):400–9. doi: 10.1111/cts.12725 (PMC7070790; doi:10.1111/cts.12725)
Supplement: Supplementary file 1 — Figures S1–S7. Table S1. Code S1, Code S2, Code S3. [file CTS-13-400-s001.pdf]

Supplementary Materials for "Using real-world data to guide ustekinumab dosing strategies for psoriasis: a prospective pharmacokinetic-pharmacodynamic study"

Figure S1: Diagram of the ustekinumab PKPD model in the current study

Figure S2: Ustekinumab clearance in patients with measurable and non-measurable ADAs (left) and individual clearances in 16 patients with ADAs (right)

Figure S3: Simulated profiles of the probability of achieving PASI 90 after 8 weekly, 12 weekly (the standard), and 16 weekly ustekinumab injections in 45 mg group (top row), 90 mg group (middle row), and escalated 90 mg group for 45 mg (bottom row)

Figure S4: Ustekinumab concentration and PASI change over time with EC50 values from the single and mixture models

Figure S5: Goodness of fit plots for observation vs. individual prediction (left panel) and conditional weighted residuals vs. time after dose (right panel), from the final ustekinumab PK model (top panel) and the final ustekinumab PKPD model (bottom panel)

Figure S6: Individual predictions versus observations for randomly selected individuals from the final ustekinumab PK model

Figure S7: Individual predictions versus observations for randomly selected individuals from the final ustekinumab PKPD model

Table S1: Parameter estimates for all patients (n=491) using two different ustekinumab PD models

Code S1: NONMEM code for the PK model of ustekinumab

Code S2: NONMEM code for the PKPD model of ustekinumab with single EC50

Code S3: NONMEM code for the PKPD model of ustekinumab with $MIXTURE

Figure S1: Diagram of the ustekinumab PKPD model in the current study (PKPD: pharmacokinetic-pharmacodynamic, k_a: absorption rate constant of ustekinumab, F: absolute bioavailability, CL: clearance, V: apparent volume of distribution, k_{in}: rate of skin lesion formation, k_{out}: rate constant of skin lesion remission, minus sign: inhibition on skin lesion progression via E(t)=(Emax*C(t))/(EC50+C(t)), E(t): inhibitory effect on skin lesion progression at time t, Emax: maximum inhibitory effect from ustekinumab, EC50: ustekinumab serum concentration at which produces 50% of the maximum inhibitory effect, C(t): ustekinumab serum concentration within the body at time t, PASI: Psoriasis Area Severity Index)

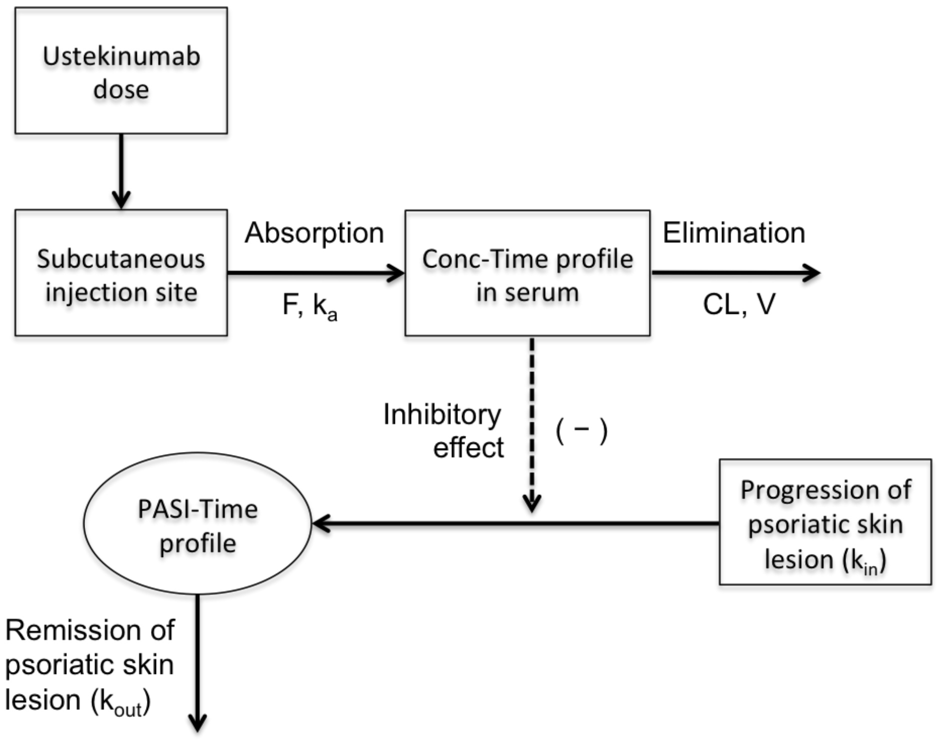

Figure S2: Boxplot of ustekinumab clearance in patients with detectable and non-detectable ADAs (left) and plot of individual clearance in 16 patients against ADA concentration (right) (USK: ustekinumab, ADA: anti-drug-antibody, AU: arbitrary unit)

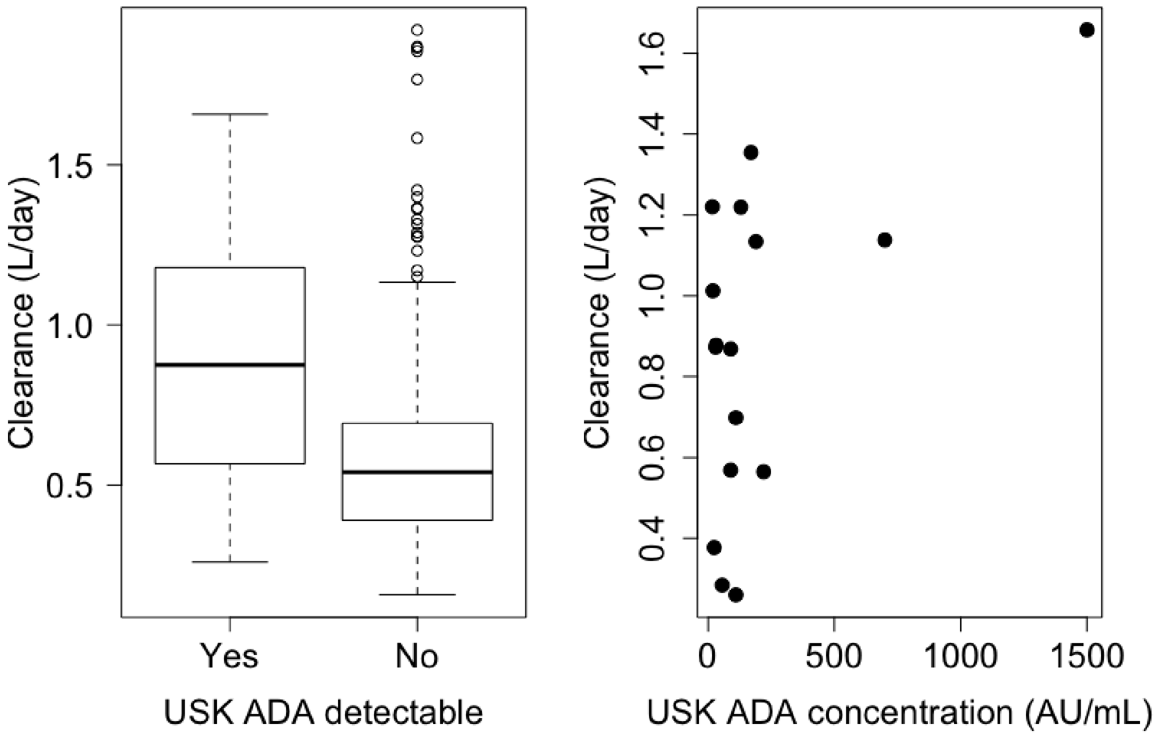

Figure S3: Simulated profiles using mixture model of the probability of achieving PASI 90 after 8 weekly, 12 weekly and 16 weekly ustekinumab injections in the patient group on 45 mg (top), on 90 mg (middle), and simulating a 90 mg dose using parameters from the patient group on 45 mg (bottom) (Group 1: using parameters from mixture group 1, Group 2: using parameters from mixture group 2, PASI: Psoriasis Area Severity Index)

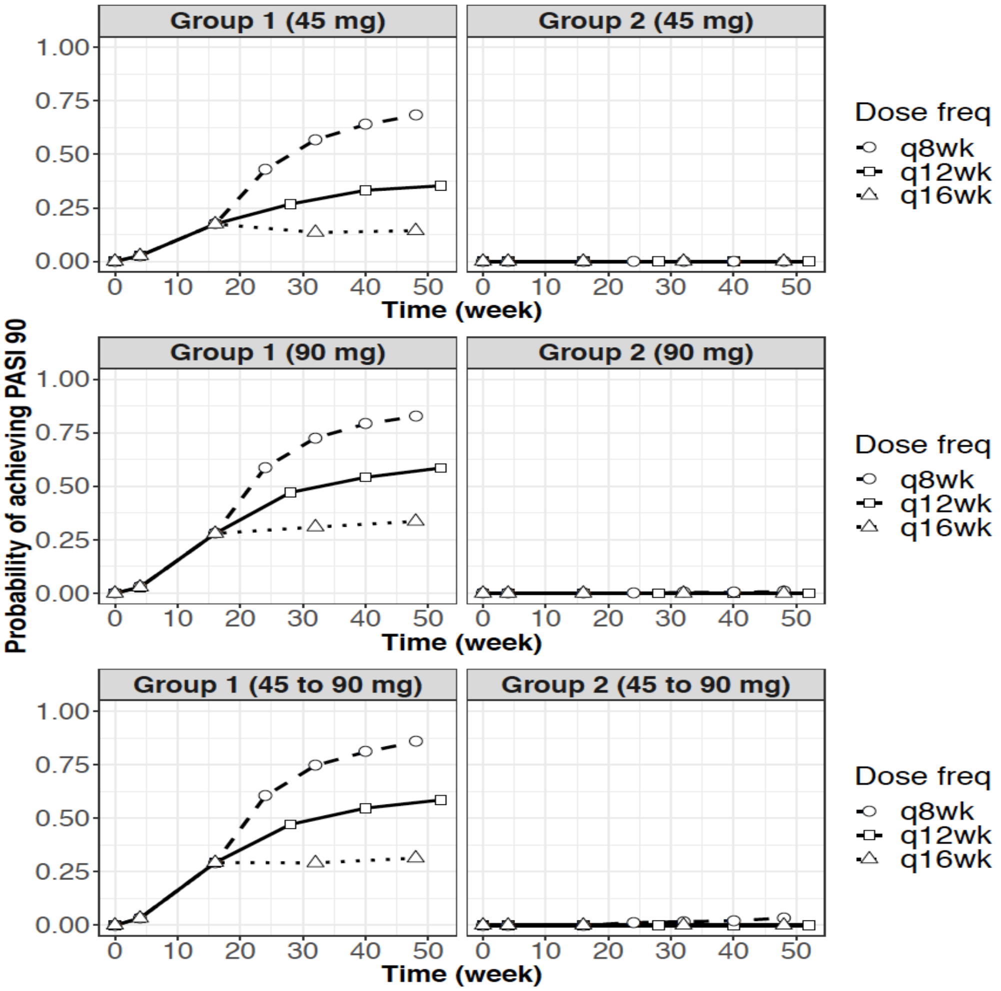

Figure S4: Ustekinumab concentration and PASI change over time with EC50 values from the single and mixture models (PASI: Psoriasis Area Severity Index, EC50: ustekinumab serum concentration at which produces 50% of the maximum inhibitory effect, green line: using EC50 value from the single model, blue line: using lower EC50 value form the mixture model, purple line: using higher EC50 value form the mixture model)

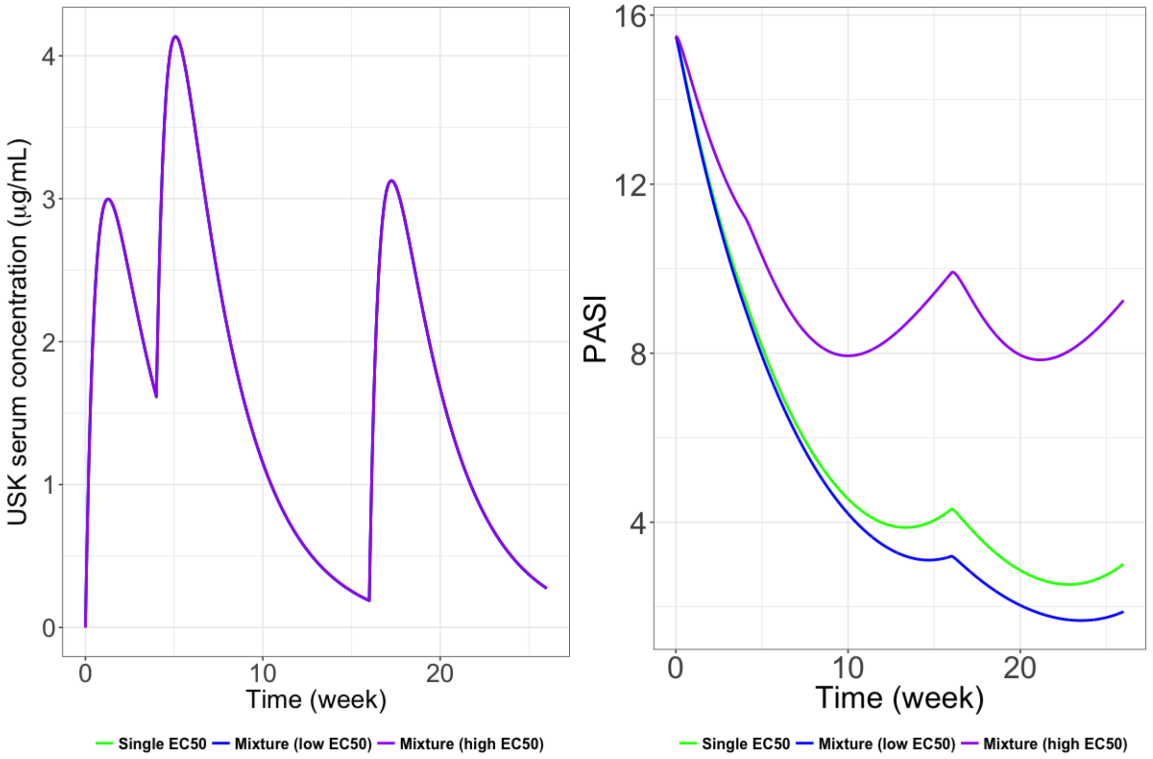

Figure S5: Goodness of fit plots for observation vs. individual prediction (left panel) and conditional weighted residuals vs. time after dose (right panel), from the final ustekinumab PK model (top panel) and the final ustekinumab PKPD model (bottom panel) (IPRED: individual prediction, CWRES: conditional weighted residuals, TAD: time after dose (day), PK: pharmacokinetic, PKPD: pharmacokinetic-pharmacodynamic, PASI: Psoriasis Area Severity Index)

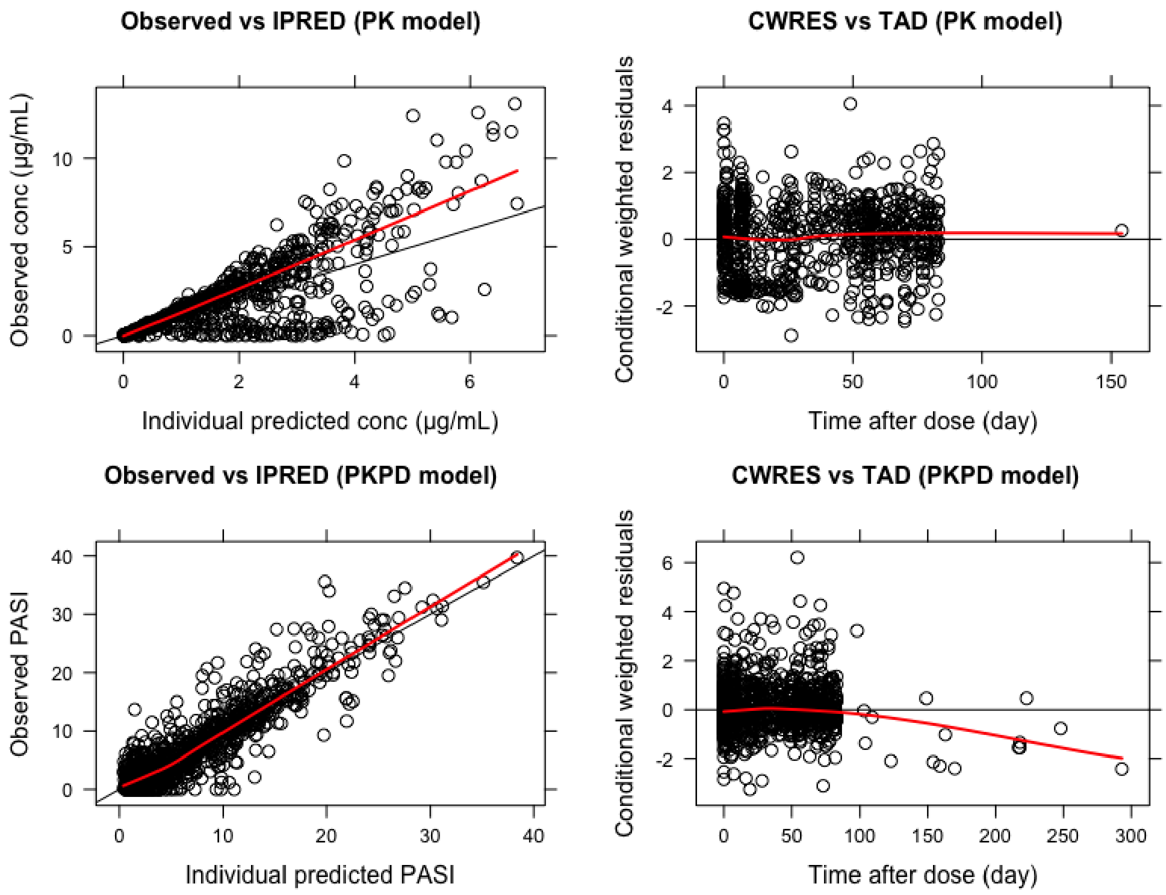

Figure S6: Individual predictions versus observations for randomly selected individuals from the final ustekinumab PK model (red line: individual prediction, blue line: population prediction, grey circle: observation, PK: pharmacokinetic)

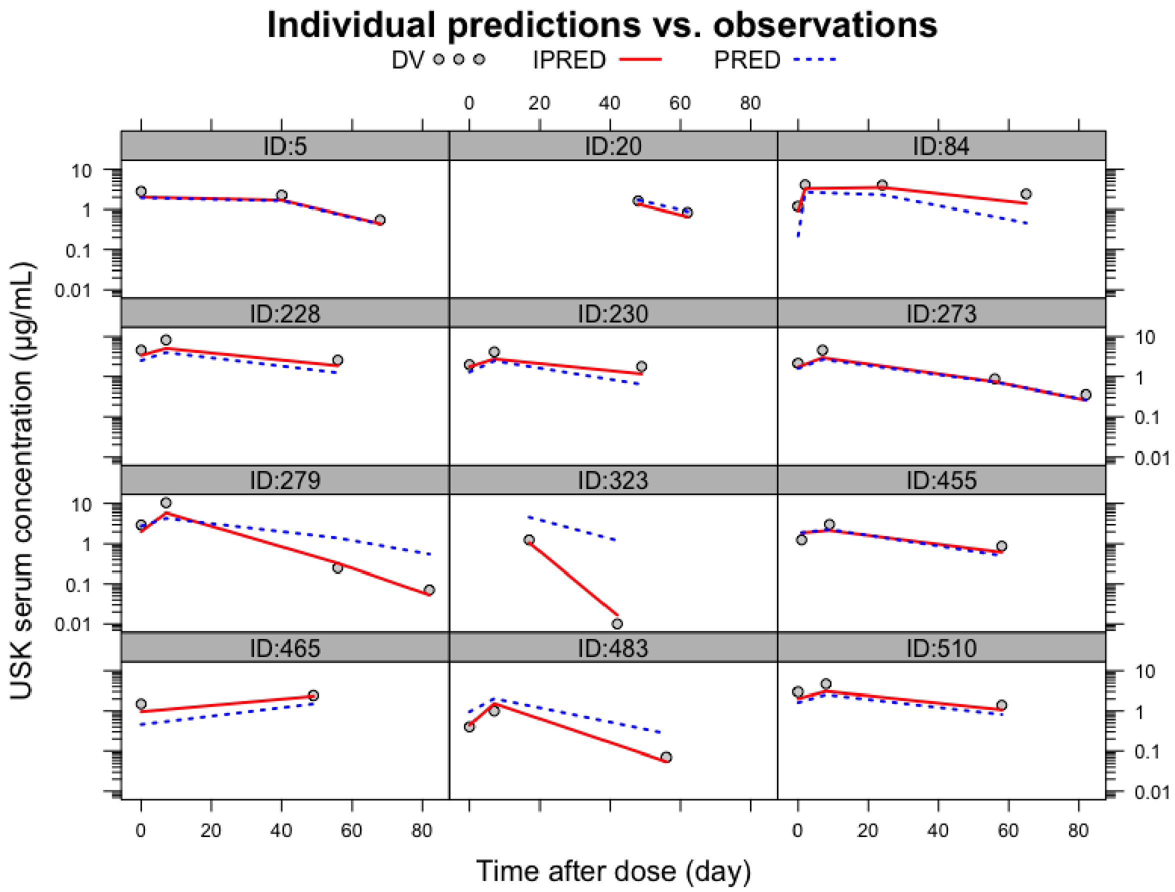

Figure S7: Individual predictions versus observations for randomly selected individuals from the final ustekinumab PKPD model (red line: individual prediction, blue line: population prediction, grey circle: observation, PKPD: pharmacokinetic-pharmacodynamic, PASI: Psoriasis Area Severity Index)

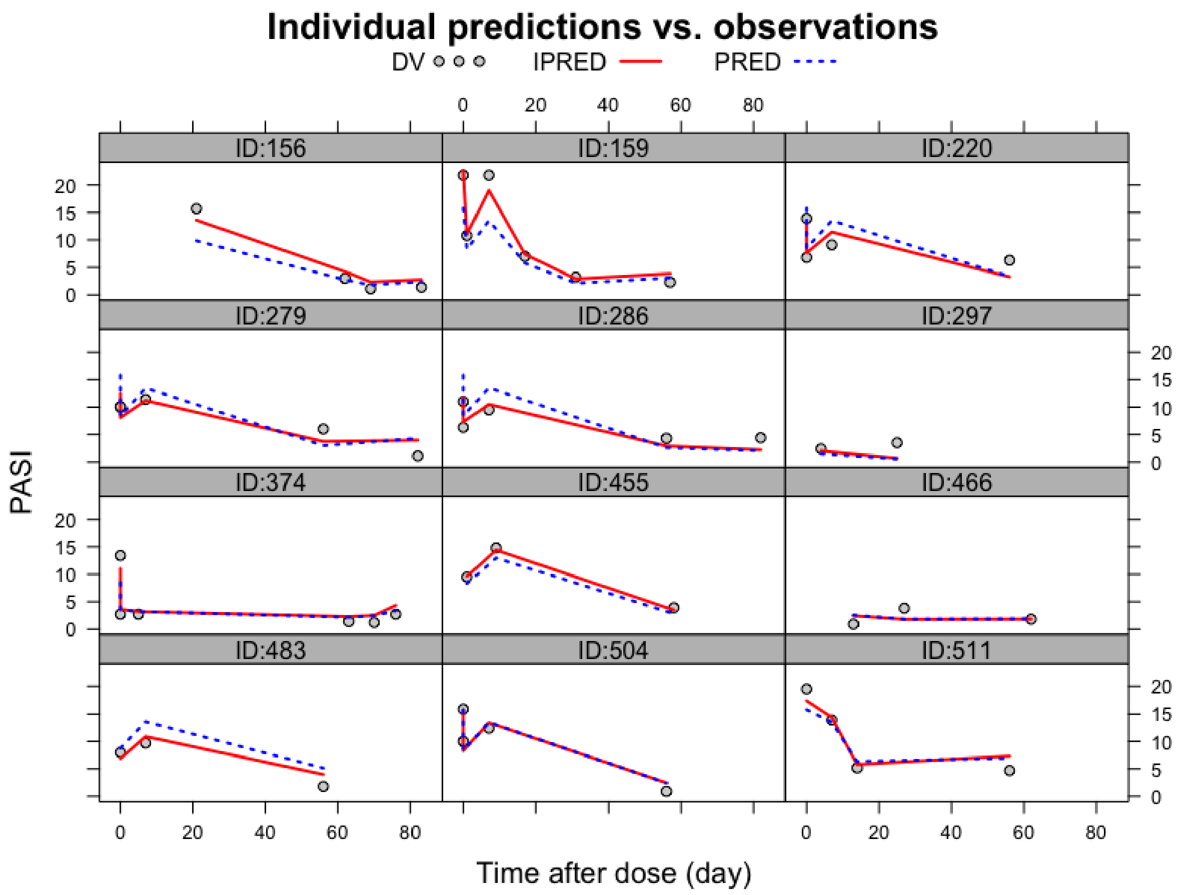

Table S1: Parameter estimates for all patients (n=491) using two different ustekinumab PD models (single model: patients treated as from a single population, mixture model: automatic stratification into two subpopulations using $MIXTURE) (PASI: Psoriasis Area Severity Index, PD: pharmacodynamic, k_{out}: elimination rate constant of skin lesions, Emax: maximum inhibition effect of ustekinumab, EC50: concentration at 50% of maximum inhibition on IL 12 and 23, BSV: between-subject variability, full-responders: ≥75% reduction from baseline, partial-responders: ≥50% and <75% reduction from baseline, non-responders: <50% reduction from baseline, delta OBJV: objective function value change from single model, RSE: relative standard error)

**Parameter (unit)**	**Estimate from single model (RSE%)**	**Estimate from mixture model (RSE%)**
baseline PASI	12.9 (4.0)	13.0 (3.9)
k_{out} (/day)	0.02 (6.1)	0.02 (3.3)
Emax	1 [fix]	1 [fix]
EC50 (µg/mL)	0.20 (11.8)	Mixture 1: 0.07 (0.6)<br>Mixture 2: 1.1 (25.4)
BSV on baseline (%)	51.4 (5.8)	50.9 (5.9)
BSV on k_{out} (%)	68.3 (6.8)	68.1 (7.6)
BSV on EC50 (%)	143.9 (8.4)	69.4 (27.2)
Additive error (%)	303.5 (1.5)	303.8 (1.4)
delta OBJV	-	-12.6

Code S1: NONMEM code for the PK model of ustekinumab

```
$PROBLEM    USK 1-CPT PK MODEL
$INPUT      ID AMT=DOSE TIME=DAY EVID DVID DV MDV ANTIUSK
CENSOREDANTIUSK BL_PASI=DROP GENDER=DROP ETHNICITY=DROP
PALMS=DROP HEIGHT WEIGHT WAIST BMI=DROP AGE=DROP
DURATION=DROP CREATININE SMOK=DROP ALCO CICL=DROP
METH=DROP METF=DROP RAMI=DROP AMLO=DROP OMEP=DROP
FOLI=DROP ASTH DEPR DYSL HYPE LIVE DIAB=DROP BIO_NAIVE
ANY_IA=DROP HLA_C_0602=DROP
$DATA      USK_NMPK_v3.csv IGNORE=@
$SUBROUTINE ADVAN2 TRANS2
$PK
;;; CLALCO-DEFINITION START
IF(ALCO.EQ.1) CLALCO = 1  ; Most common
IF(ALCO.EQ.0) CLALCO = ( 1 + THETA(7))
IF(ALCO.EQ.-99) CLALCO = 1  ; Missing data
;;; CLALCO-DEFINITION END

;;; CLWAIST-DEFINITION START
IF(WAIST.EQ.-99) THEN
   CLWAIST = 1
ELSE
   CLWAIST = ((WAIST/104)**THETA(6))
ENDIF
;;; CLWAIST-DEFINITION END

;;; CLCREATININE-DEFINITION START
IF(CREATININE.EQ.-99) THEN
   CLCREATININE = 1
ELSE
   CLCREATININE = ((CREATININE/78)**THETA(5))
ENDIF
;;; CLCREATININE-DEFINITION END

;;; CLBIO_NAIVE-DEFINITION START
IF(BIO_NAIVE.EQ.0) CLBIO_NAIVE = 1  ; Most common
IF(BIO_NAIVE.EQ.1) CLBIO_NAIVE = ( 1 + THETA(4))
;;; CLBIO_NAIVE-DEFINITION END

;;; CL-RELATION START
CLCOV=CLBIO_NAIVE*CLCREATININE*CLWAIST*CLALCO
;;; CL-RELATION END

 F1 = 1
 KA = THETA(1)
TVCL = THETA(2)*(WEIGHT/70)**0.75
TVCL = CLCOV*TVCL
CL = TVCL*EXP(ETA(1))
TVV = THETA(3)*(WEIGHT/70)**1
V = TVV*EXP(ETA(2))


;--- TIME AFTER DOSE
IF(EVID==1) TM=TIME
```

```
IF(EVID==1) TAD=0
IF(EVID/=1) TAD=TIME-TM

$ERROR
IPRED = A(2)/V
Y = IPRED*(1 + EPS(1)) + EPS(2)
PROP = SQRT(SIGMA(1,1))*IPRED
ADD = SQRT(SIGMA(2,2))
SD = SQRT(PROP*PROP + ADD*ADD)
IRES = DV-IPRED
IWRES = IRES/SD

$THETA  (0,0.229631) ; KA (/day)
(0,0.439693) ; CL (L/day)
(0,10.2307) ; V (L)
$THETA  (-1,-0.127533,5) ; CLBIO_NAIVE1
$THETA  (-100,-0.354595,100000) ; CLCREATININE1
$THETA  (-100,0.837662,100000) ; CLWAIST1
$THETA  (-1,0.144836,5) ; CLALCO1
$OMEGA  BLOCK(2)
 0.200201
 0.0601176 0.132511
$SIGMA  0.376028
 4.44216E-05

$ESTIMATION MAXEVAL=9999 METHOD=1 INTERACTION POSTHOC NOHABORT PRINT=5
$COVARIANCE UNCONDITIONAL PRINT=E
$TABLE      ID TIME AMT DVID PRED IPRED CWRES TAD NOPRINT ONEHEADER
FILE=sdtab10
$TABLE      ID KA CL V ETA1 ETA2 NOPRINT NOAPPEND ONEHEADER FILE=patab10
$TABLE      ID ANTIUSK CENSOREDANTIUSK NOPRINT NOAPPEND ONEHEADER
FILE=cotab10
$TABLE      ID NOPRINT NOPRINT NOAPPEND ONEHEADER FILE=catab10
```

Code S2: NONMEM code for the PKPD model of ustekinumab—single model

```
$PROBLEM  USK PKPD TURNOVER WITH IPP + SINGLE
$INPUT    ID AMT=DOSE TIME=DAY EVID DVID DV MDV ANTIUSK CENSOREDANTIUSK
BL_PASI GENDER ETHNICITY PALMS HEIGHT WEIGHT WAIST BMI AGE DURATION
CREATININE SMOK ALCO CICL METH METF OMEP FOLI ASTH DEPR DYSL HYPE LIVE
DIAB BIO_NAIVE ANY_IA HLA_C_0602 IKA ICL IV RESDER_CAT
$DATA     USK_NMPKPD_Year_IPP_BSL_v4.csv IGNORE=@ IGNORE=(DVID.EQ.1,
DVID.GT.2) IGNORE=(BL_PASI<10, ID.EQ.350)
$SUBROUTINE ADVAN6 TOL=6
$MODEL
COMP=(DOSE)
COMP=(CENTRAL)
COMP=(PASI)
$PK
F1 = 1
KA = IKA
CL = ICL
V  = IV
KE = CL/V
BSL = THETA(1)*EXP(ETA(1))
KOUT = THETA(2)*EXP(ETA(2))
EMAX = THETA(3)
IC50 = THETA(4)*EXP(ETA(3))
KIN = BSL*KOUT
A_0(3) = BSL

;--- Time after dose
IF(EVID==1) TM=TIME
IF(EVID==1) TAD=0
IF(EVID/=1) TAD=TIME-TM

$DES
DADT(1) = -KA*A(1)
DADT(2) = KA*A(1) - KE*A(2)
CONC = A(2)/V
DG = EMAX*CONC/(IC50+CONC)
DADT(3) = KIN*(1-DG)-KOUT*A(3)

$ERROR
IPRED = A(3)
Y = IPRED + EPS(1)
ADD = SQRT(SIGMA(1,1))
SD = ADD
IRES = DV-IPRED
IWRES = IRES/SD

$THETA
(0, 15)        ;BSL
(0, 0.1)       ;KOUT (per DAY)
1 FIX          ;EMAX
(0, 10)        ;IC50 (ug/mL)
$OMEGA
0.1
0.1
```

```
10
$SIGMA
0.1

$ESTIMATION MAXEVAL=9999 METHOD=1 INTERACTION POSTHOC NOHABORT PRINT=5
$COV UNCONDITIONAL PRINT=E MATRIX=S
$TABLE ID TIME DVID IPRED IWRES CWRES TAD EVID NOPRINT ONEHEADER
FILE=sdtab43
$TABLE ID BSL KOUT EMAX IC50 ETA1 ETA2 ETA3 NOPRINT NOAPPEND ONEHEADER
FILE=patab43
$TABLE ID RESDER_CAT ANTIUSK BL_PASI HEIGHT WEIGHT WAIST BMI AGE DURATION
CREATININE IKA ICL IV NOPRINT NOAPPEND ONEHEADER FILE=cotab43
$TABLE ID RESDER_CAT CENSOREDANTIUSK GENDER ETHNICITY PALMS SMOK ALCO
CICL METH METF OMEP FOLI ASTH DEPR DYSL HYPE LIVE DIAB BIO_NAIVE ANY_IA
HLA_C_0602 NOPRINT NOAPPEND ONEHEADER FILE=catab43
```

Code S3: NONMEM code for the PKPD model of ustekinumab—mixture model

```
$PROBLEM  USK PKPD TURNOVER WITH IPP + $MIXURE
$INPUT    ID AMT=DOSE TIME=DAY EVID DVID DV MDV ANTIUSK CENSOREDANTIUSK
BL_PASI GENDER ETHNICITY PALMS HEIGHT WEIGHT WAIST BMI AGE DURATION
CREATININE SMOK ALCO CICL METH METF OMEP FOLI ASTH DEPR DYSL HYPE LIVE
DIAB BIO_NAIVE ANY_IA HLA_C_0602 IKA ICL IV RESDER_CAT
$DATA     USK_NMPKPD_Year_IPP_BSL_v4.csv IGNORE=@ IGNORE=(DVID.EQ.1,
DVID.GT.2) IGNORE=(BL_PASI<10, ID.EQ.350)
$SUBROUTINE ADVAN6 TOL=6
$MODEL
COMP=(DOSE)
COMP=(CENTRAL)
COMP=(PASI)
$PK
F1 = 1
KA = IKA
CL = ICL
V  = IV
KE = CL/V
BSL = THETA(1)*EXP(ETA(1))
KOUT = THETA(2)*EXP(ETA(2))
EMAX = THETA(3)
KIN = BSL*KOUT
A_0(3) = BSL
; MIXTURE MODEL
EST = MIXEST
IF (MIXNUM.EQ.1) THEN
  C50 = THETA(4)
ELSE
  C50 = THETA(5)
ENDIF
IC50 = C50*EXP(ETA(3))

;--- Time after dose
IF(EVID==1) TM=TIME
IF(EVID==1) TAD=0
IF(EVID/=1) TAD=TIME-TM

$DES
DADT(1) = -KA*A(1)
DADT(2) = KA*A(1) - KE*A(2)
CONC = A(2)/V
DG = EMAX*CONC/(IC50+CONC)
DADT(3) = KIN*(1-DG)-KOUT*A(3)

$ERROR
IPRED = A(3)
Y = IPRED + EPS(1)
ADD = SQRT(SIGMA(1,1))
SD = ADD
IRES = DV-IPRED
IWRES = IRES/SD

$MIX
```

```
P(1) = THETA(6)
P(2) = 1-THETA(6)
NSPOP = 2

$THETA
(0, 15)        ;BSL
(0, 0.1)       ;KOUT (per DAY)
1 FIX          ;EMAX
(0, 0.5)       ;C501 (ug/mL)
(0, 1)         ;C502 (ug/mL)
(0, 0.2)       ;P(1)
$OMEGA
0.1
0.1
5
$SIGMA
0.1

$ESTIMATION MAXEVAL=9999 METHOD=1 INTERACTION POSTHOC NOHABORT PRINT=5
$COV UNCONDITIONAL PRINT=E MATRIX=S
$TABLE ID TIME DVID IPRED IWRES CWRES TAD EVID NOPRINT ONEHEADER
FILE=sdtab44
$TABLE ID BSL KOUT EMAX IC50 ETA1 ETA2 ETA3 NOPRINT NOAPPEND ONEHEADER
FILE=patab44
$TABLE ID RESDER_CAT ANTIUSK BL_PASI HEIGHT WEIGHT WAIST BMI AGE DURATION
CREATININE IKA ICL IV NOPRINT NOAPPEND ONEHEADER FILE=cotab44
$TABLE ID RESDER_CAT CENSOREDANTIUSK GENDER ETHNICITY PALMS SMOK ALCO
CICL METH METF OMEP FOLI ASTH DEPR DYSL HYPE LIVE DIAB BIO_NAIVE ANY_IA
HLA_C_0602 NOPRINT NOAPPEND ONEHEADER FILE=catab44
```
